# Lipid-coated mesoporous silica nanoparticles for anti-viral applications via delivery of CRISPR-Cas9 ribonucleoproteins

**DOI:** 10.1038/s41598-023-33092-4

**Published:** 2023-04-27

**Authors:** Annette E. LaBauve, Edwin A. Saada, Iris K. A. Jones, Richard Mosesso, Achraf Noureddine, Jessica Techel, Andrew Gomez, Nicole Collette, Michael B. Sherman, Rita E. Serda, Kimberly S. Butler, C. Jeffery Brinker, Joseph S. Schoeniger, Darryl Sasaki, Oscar A. Negrete

**Affiliations:** 1 Department of Biotechnology and Bioengineering, Sandia National Laboratories, Livermore, USA; 2 Department of Systems Biology, Sandia National Laboratories, Livermore, USA; 3 Chemical and Biological Engineering, University of New Mexico, Albuquerque, USA; 4 Center for Micro-Engineered Materials, University of New Mexico, Albuquerque, USA; 5 Department of Active Ceramics Value Stream, Sandia National Laboratories, Albuquerque, USA; 6 Sealy Center for Structural Biology and Molecular Biophysics, University of Texas Medical Branch, Galveston, USA; 7 Department of Internal Medicine, Health Sciences Center, University of New Mexico, Albuquerque, USA; 8 Department of Molecular and Microbiology, Sandia National Laboratories, Albuquerque, USA; 9 Advanced Materials Laboratory, Sandia National Laboratories, Albuquerque, USA; 10 Present Address: Biotechnology and Biosciences Division, Physical and Life Sciences Directorate, Lawrence Livermore National Laboratory, Livermore, USA

**Keywords:** Biomaterials, Cell delivery, Nanobiotechnology, Protein delivery, Infection

## Abstract

Emerging and re-emerging viral pathogens present a unique challenge for anti-viral therapeutic development. Anti-viral approaches with high flexibility and rapid production times are essential for combating these high-pandemic risk viruses. CRISPR-Cas technologies have been extensively repurposed to treat a variety of diseases, with recent work expanding into potential applications against viral infections. However, delivery still presents a major challenge for these technologies. Lipid-coated mesoporous silica nanoparticles (LCMSNs) offer an attractive delivery vehicle for a variety of cargos due to their high biocompatibility, tractable synthesis, and amenability to chemical functionalization. Here, we report the use of LCMSNs to deliver CRISPR-Cas9 ribonucleoproteins (RNPs) that target the Niemann–Pick disease type C1 gene, an essential host factor required for entry of the high-pandemic risk pathogen Ebola virus, demonstrating an efficient reduction in viral infection. We further highlight successful in vivo delivery of the RNP-LCMSN platform to the mouse liver via systemic administration.

## Introduction

Emerging and re-emerging viruses present substantial risks to global health and economic stability, as evidenced by the 2014–2016 Ebola virus (EBOV) epidemic in western Africa^1^, as well as the SARS-CoV-2 pandemic^2^. The viruses encompassed by this group span a breadth of viral families from coronaviruses to filoviruses, bunyaviruses, and alphaviruses, and includes as-of-yet unidentified viral pathogens. This presents a unique difficulty in developing therapeutics and vaccines to mitigate impacts of future epidemics and pandemics.

While successful vaccines were developed during the recent EBOV epidemic and SARS-CoV-2 pandemic, their efficacy was limited to specific viral strains or variants. For example, the current vesicular stomatitis virus (VSV)-based vaccine (VSV-EBOV) has shown good efficacy against the EBOV Zaire strain^3^. However, this vaccine could not be deployed during the 2022 EBOV Sudan outbreak in Uganda due to incomplete cross protection across viral strains^4^. Additionally, traditional therapeutic agents targeting specific pathogens of interest face issues in the rapid emergence of drug resistance. To help combat emerging and re-emerging viruses, new methods in medical countermeasure development are needed.

Host-directed therapeutic approaches are emerging as potent options to prepare broad-spectrum countermeasures with a high genetic barrier against resistance^5,6^. For example, the entry receptor for EBOV is known (Niemann–Pick disease type C1 protein—NPC1) and knock-down of this receptor blocks viral entry and infection of multiple filoviruses, including EBOV, Marburg virus, and Sudan virus^7^. Moreover, mutations in the EBOV genome to circumvent use of the NPC1 receptor have never been described. Safely inhibiting the interaction between EBOV and the NPC1 protein by host gene suppression would therefore not only serve as an effective treatment for EBOV, but also for Marburg virus, Sudan virus, and most likely other undiscovered bat-borne filoviruses.

CRISPR-based gene editing technologies have the potential to make impactful advances for infectious diseases^8^. Not long after the discovery of CRISPR-based genome editors, their applications to the prevention and treatment of HIV-1 infection was demonstrated in cell-based studies. Specifically, CRISPR/Cas9 technology was shown to target cellular co-factors (e.g. CCR5), reducing HIV-1 infection, and to target the HIV-1 proviral genome to clear the provirus^9^. More recently, newer technologies based on RNA-targeting CRISPR systems such as Cas13d have shown that reducing lung Cathepsin L host protein expression can block SARS-Cov2 infection in mice^10^. While these studies highlight promising approaches to countermeasure development against infectious diseases, further advancement of CRISPR-based anti-viral therapeutics will require additional exploration of new vehicles for the safe and effective delivery of CRISPR cargos to target tissues.

Multiple delivery options are available for administration of Cas enzymes along-side guide RNA (gRNA) sequences, including viral delivery, plasmid transfection, RNA transfection, or transfection of an externally produced Cas protein as a ribonucleoprotein (RNP) complex. Of these approaches, delivery of the Cas9 DNA-editing enzyme as a RNP has several advantages, including a high editing efficacy with low off-target effects due to transient exposure of the target genome to the Cas protein^11^. While viral delivery methods also exhibit high editing efficacy, they can result in insertional mutagenesis, host-immune responses against either the viral delivery vector or cells transformed to express the Cas enzyme, and exhibit higher off-target rates due to prolonged exposure of the target genome to the Cas enzyme^12,13^. Among non-viral delivery methods for CRISPR cargoes, lipid nanoparticles (LNPs) have recently gained increased attention in part due to their safety profile and recently expanded use in vaccine development. While LNPs have been formulated to deliver CRISPR-RNP complexes in vivo, their editing efficiency has been much lower than LNP mediated delivery of gRNA with mRNA-encoded Cas9^14–16^. Comparatively, delivery of CRISPR-RNP has distinct advantages over CRISPR-RNA that warrant continued optimization of RNP delivery vehicles to improve in vivo efficacy. For example, while the Cas9 mRNA and gRNA in CRISPR-RNA systems are simultaneously delivered to a cell, the timing of mRNA translation creates a lag between delivery and RNP assembly during which the gRNA is susceptible to degradation. Although chemical modifications aimed at increasing gRNA stability and prolonging Cas9/gRNA activity have been introduced, these alterations may increase the chances of off-target effects or immunogenicity complications.

Of the formulations successfully tested for RNP delivery, solid nanoparticles are attractive because they allow for a wide range of possible chemical modifications and attachment methods. Specifically, lipid-coated mesoporous silica nanoparticles (LCMSNs) have intriguing properties as delivery vehicles for CRISPR-RNP. LCMSNs have been used in drug delivery systems to improve drug stability and solubility, protect cargo, target specific tissues, and enhance drug circulation time and controlled release^17–19^. In addition, mesoporous silica nanoparticle (MSN) pore size, pore chemistry and overall particle size can be individually tailored, allowing for efficient and high-capacity loading of disparate cargoes, or even the co-delivery of drug and protein combinations^20–22^. Previously, our group demonstrated successful editing using LCMSNs, achieving approximately 70% delivery of RNP payload resulting in 10% editing in a reporter cell line, and localized in vivo delivery via stereotaxic injection in an Ai9-tdTomato reporter mouse resulting in approximately 10% editing in the mouse brain^23^. However, use of LCMSNs for anti-viral applications via delivery of CRISPR-RNP will require further exploration of enhanced endogenous gene targeting and systemic in vivo delivery to match the tissue tropism of a virus and effectively reduce or block cell infection. Optimization of pore size and/or pore chemistry to enhance endogenous gene editing has yet to be studied. Additionally, as the liver is the predominant target of infection in many animal models of emerging viruses, including of EBOV, extending the tissue targets of the RNP-LCMSN platform to liver delivery would benefit applications in anti-viral development^24,25^.

In this study, we demonstrate successful delivery of CRISPR-Cas9 RNPs targeting the NPC1 host gene necessary for cell entry of EBOV using LCMSNs, as well as demonstrating successful liver targeting in a mouse reporter model. These proof-of-concept studies support the role of host-directed antiviral therapies, and potential for future use of CRISPR-mediated methods when delivery vehicles are combined with safe effectors that temporarily regulate host gene expression.

## Results and discussion

### Section 1: selection of the optimal MSN core for RNP loaded LCMSN colloidal stability and cellular delivery

**Figure 1:**
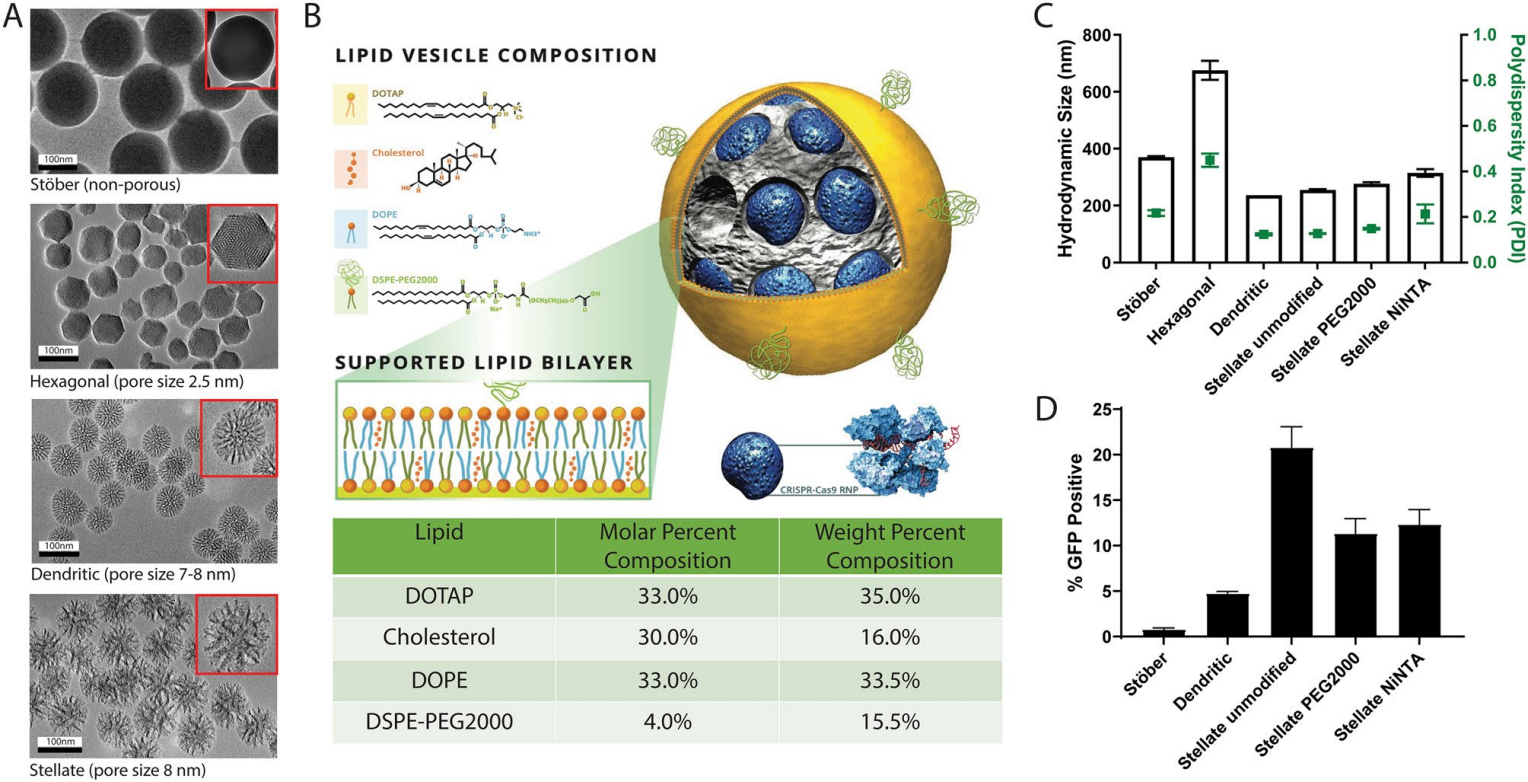
MSN screening identifies an optimal LCMSN formulation for cellular delivery of CRISPR RNP. (**A**) TEM images of 4 non-lipid coated MSN particle types: Stӧber, hexagonal, dendritic, and stellate morphologies. (**B**) Schematic of the LCMSN platform highlighting the composition of lipid coating in the DCD3330 formulation. (**C**) Hydrodynamic size (black) and polydispersity index (green) measurements of RNP loaded LCMSNs determined by DLS. (**D**) Screening of RNP-LCMSN formulations using a reporter cell line (Fig. S1).

MSNs can be formulated in a variety of different configurations via the sol–gel process and the pore size of MSNs has played a critical role for the loading and release of model proteins such as bovine serum albumin and RNase A^21^. Taking into account that Cas9 protein from *Streptococcus pyogenes* in complex with gRNA has an overall negative charge with 10 nm × 10 nm × 50 nm dimensions^26^, we began by evaluating MSN particles with varying morphologies and pore sizes to assess their ability to optimally load CRISPR-Cas9 RNP into LCMSNs with positively charged lipids, and deliver colloidally stable formulations to cells for gene editing applications. Specifically, MSNs with plain (Stӧber), hexagonal, stellate, and dendritic particle morphologies and pore sizes (non-porous and pore diameters centered at 2 nm, 7 nm, and 10 nm) were chosen (Fig. 1A). These nanoparticle syntheses were reproduced from our previous work^17,27^. Each MSN core type was used to prepare RNP loaded LCMSNs (RNP-LCMSNs) with an optimized liposome formulation consisting of 33 mol% DOTAP, 33 mol% DOPE, 30 mol% cholesterol, and 4 mol% DSPE-PEG2000, called DCD3330 (Fig. 1B), which was identified upon screening various lipid formulations containing variable amounts of the aforementioned lipids. Previous studies have demonstrated that specific lipids within this optimized formulation offer enhanced cellular uptake due to the DOTAP-borne local positive charge^28^ and endosomal escape upon endocytosis^29,30^. To determine the optimal arrangement for the RNP-LCMSNs, we compared hydrodynamic size and polydispersity index values for each of the four morphologies along with additional surface-chemically modified stellate particles (Fig. 1C). These surface modifications included a PEG2000 coating and a disulfide-bridged Nickel NTA (NiNTA) coating. The PEG2000 coating was chosen to block native RNP binding to the silica surface, theoretically allowing for crowding of soluble RNP between the lipid coat and the PEG2000-silica nanoparticle, which may more freely release RNP intracellularly. Conversely, the chemical attachment of disulfide-bridged NiNTA was chosen to allow for specific binding of His-tagged Cas9, and controlled release upon intracellular exposure to cytosolic glutathione^31,32^. Stӧber, stellate, and dendritic particle formulations all met the size requirements for incorporation of RNP while maintaining capacity for lipid coating. These particles also offered low polydispersity index and high colloidal stability compared to the small-pore hexagonal based RNP-LCMSNs (Fig. 1C). Both stellate variants showed a slightly larger RNP-LCMSN, in the 250–300 nm size range, but maintained low polydispersity index values of ≤ 0.2, indicating a homogeneous size distribution of nanoparticles (Fig. 1C).

The selected RNP-LCMSNs were then tested using a red fluorescent protein (RFP)/ GFP CRISPR reporter system^33^ in which successful delivery of the CRISPR-Cas9 RNP and subsequent editing as measured by InDel formation at the target site would result in GFP expression (Fig. S1). For these assays the hexagonal MSN was excluded as this morphology showed large particle sizes in our initial screenings. Here, we found that induction of InDels by stellate DCD3330 LCMSN increased over time up to 72 h (Fig. S1). To examine the impact of particle morphology on CRISPR-mediated gene editing efficacy, we have used the same mass of particles amongst all assessed samples (40 μg). Importantly, the unmodified stellate LCMSN resulted in the best editing in this reporter cell line (21%), compared to 1%-12% editing among the other particle morphologies (Fig. 1D), highlighting a twofold increase improvement over previously reported RNP-LCMSN formulations^23^. As such, the unmodified stellate MSN-based RNP-LCMSNs were selected for further investigation. Synthesis of stellate MSN yielded between 500 and 800 mg of MSN, which are shelf stable when stored in ethanol for over a year and exhibited defined pore sizes able to accommodate the desired RNP payload (Fig. S2).

### Section 2: comprehensive characterization of the selected RNP-LCMSN formulation

**Figure 2:**
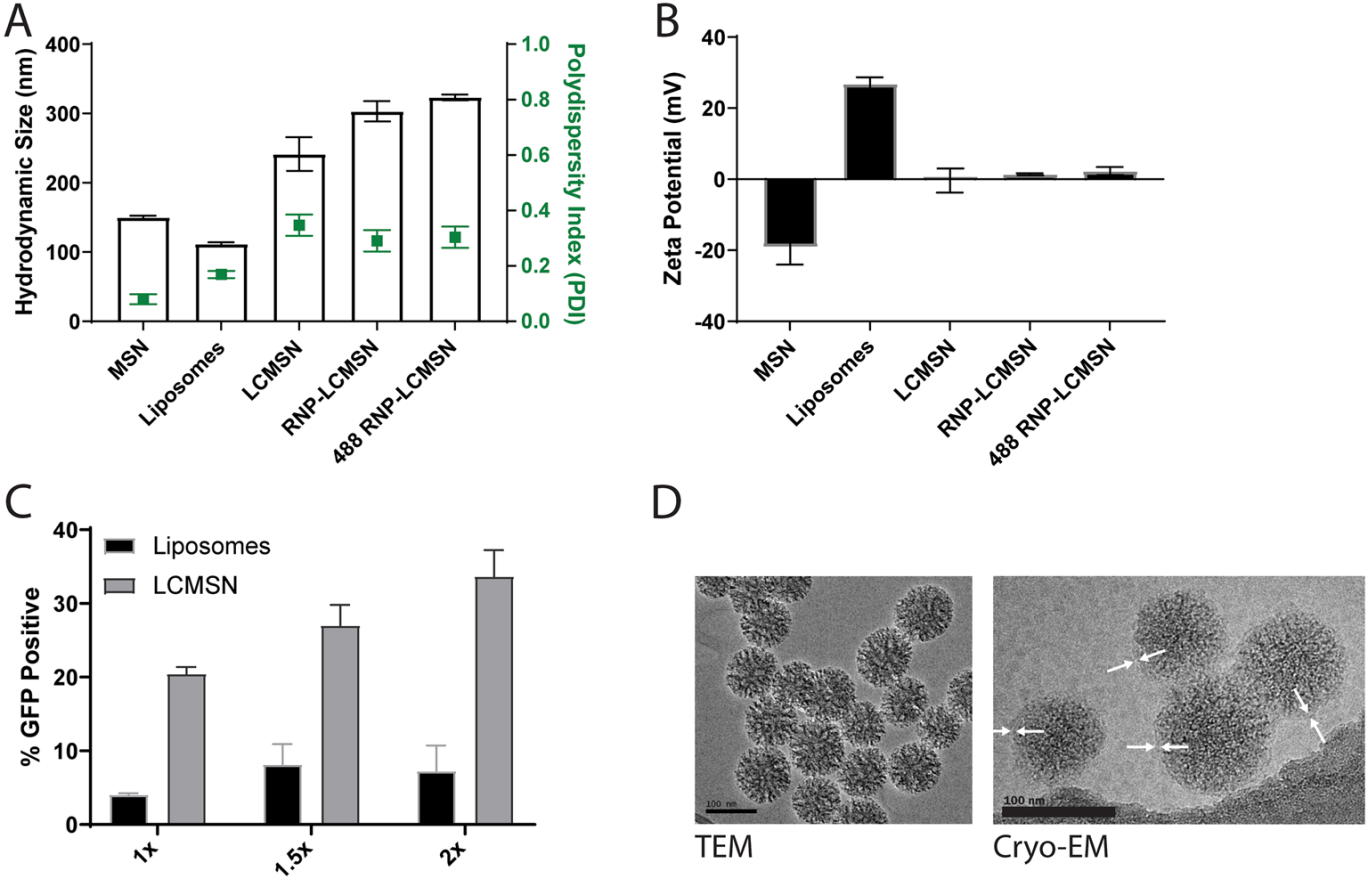
Formation analysis, dose-dependent editing, and ultrastructure characterization of the down-selected RNP-LCMSN. (**A**) Hydrodynamic size and polydispersity index values for nanoparticle and liposome formulations. (**B**) Zeta-potential of nanoparticle and liposome formulations. (**C**) Editing efficacy of LCMSN and liposomes in a GFP-reporter assay (Figure S1). 1x = 5.5 µg RNP = 40 µγ MSN, 1.5 = 8.4 µg RNP = 60 µγ MSN, and 2x = 11.2 µg RNP = 80 µγ MSN. (**D**) TEM and Cryo-EM images of DCD3330 RNP-LCMSNs.

After identifying an RNP-LCMSN formulation suitable for further development and characterization, we proceeded to understand the nanoparticle size, uniformity and charge changes observed throughout the formation of the selected RNP-LCMSN construct. To accomplish this, we performed DLS and zeta-potential measurements on uncoated MSNs, DCD3330 liposomes, LCMSNs, RNP-loaded LCMSNs, and LCMSNs loaded with a fluorescently tagged RNP (488 RNP-LCMSN) (Fig. 2A, B). The LCMSN showed an increase in size over the uncoated MSNs, and a slight further increase in size was seen with loading of the LCMSNs. Notably, the 488 RNP-LCMSN did not substantially increase the hydrodynamic size, polydispersity index, or zeta potential of the particles (Fig. 2A, B), constituting a labeled RNP-LCMSN formulation for intracellular delivery tracking studies that theoretically mimics the original unlabeled construct.

To demonstrate that our lipid coating served to stabilize the RNP-loaded MSNs, we measured particle size of RNP-MSNs and RNP-LCMSNs over time. The DCD3330-coating on RNP-loaded MSNs provided a strong stabilizing effect, wherein LCMSN particles were size stable for over a week (Fig. S2). In contrast, the RNP-complexed MSNs without lipid-coating failed to form a monodisperse population, and quickly formed microparticle-sized aggregates. Importantly, we evaluated the in vitro editing capacity of our LCMSN made with DCD3330 compared to their DCD3330 liposome counterparts, and the LCMSNs exhibited a five-fold increase in editing efficiency when compared to liposomes at the 5.6 μg (1x), 8.4 μg (1.5x), and 11.2 μg (2x) amounts of loaded Cas9 RNP (Fig. 2C). Cryo-EM of the particles showed complete lipid coating of the RNP loaded nanoparticles (Fig. 2D, indicated by arrows).

**Figure 3:**
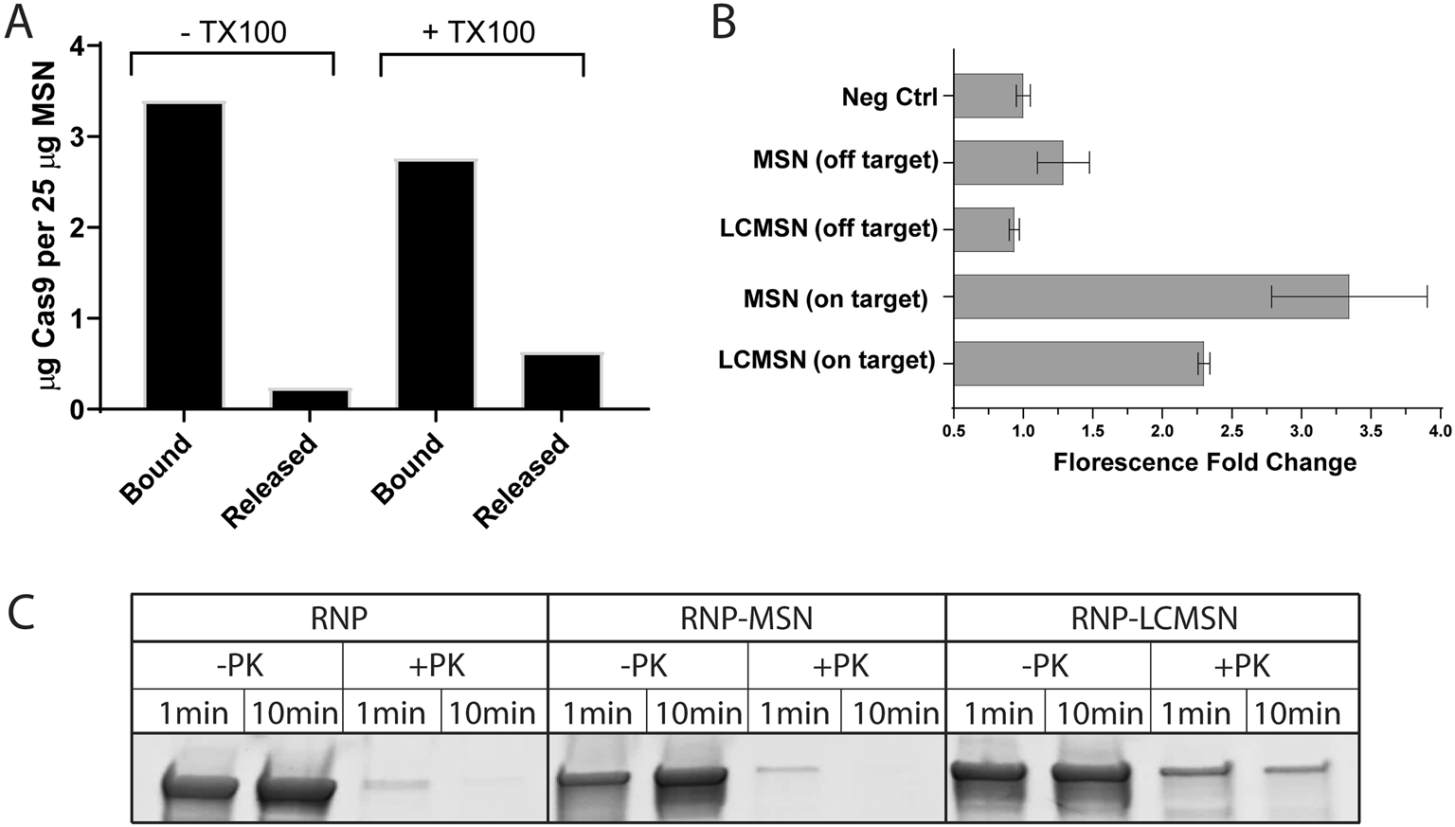
Characterization of RNP binding, release, activity, and protection in LCMSN formulations. (**A**) RNP loaded LCMSNs were treated with (+) and without (−) Triton X-100 to determine the amount of RNP released upon lipid coating disruption. Cas9 protein was quantified through densiometric analysis of SDS-PAGE visualized by Coomassie stain (Fig. S3) and shown as representative data. (**B**) A FRET-based Cas9 activity assay^33^ measured the activity of Cas9-RNP when loaded on MSNs or LCMSNs, and in the presence of active (on-target) or negative control (off-target) gRNAs. (**C**) Free RNP, RNP loaded MSN without (MSN-RNP) and with lipid coatings (RNP-LCMSN) were exposed to proteinase K (+ PK) for 1–10 min. Cas9 protein was visualized by gel electrophoresis and Coomassie staining.

The lipid layer is also hypothesized to protect and harness RNP cargo. To measure retention of RNP to particles, a protein solubility and release assay was used, wherein RNP-loaded LCMSNs (140 μg of Cas9/1 mg particle) were treated with or without triton X-100 for 4 h. The amount of protein released into the supernatant after treatment and incubation was assessed via SDS-PAGE analyses, with the non-treated sample showing approximately 94% bound with 6% released, while the Triton X treated sample showed approximately 82% bound and 18% released. Nearly three times more protein was released in the triton X-100 treated samples (25 µg per mg of MSN, compared to 10 µg of Cas9 per mg of lipid-free MSN) (Figs. 3A, S3), indicating that the lipid coating aids in retention of RNP by the LCMSNs.

Additionally, thermal gravimetric analysis (TGA) was used to measure the stability of RNP-LCMSNs as a function of temperature and confirmed the release and disassembly of both DCD3330 lipid bilayer and RNP from RNP-LCMSNs upon heating (Fig. S4). The TGA absolute weight loss profile showed that LCMSNs underwent a ~ 55% weight loss that was attributed to degradation of the DCD3330 lipid bilayer, and ~ 60% weight loss for RNP-LCMSNs that was attributed to degradation of both RNP and lipids.

As RNP and the DCD3330 lipids exhibit opposite charges, there remains the likelihood for interaction and binding to both sides of the lipid membrane. To better understand the degree of RNP accessibility, we utilized two assays focusing on the RNP cargo. The first assay aimed to determine the catalytic activity retention of RNP when formulated in LCMSNs. We adapted an in vitro Cas9 FRET assay^33^, and generated RNP loaded MSN and LCMSN using active and control gRNA sequences (Fig. 3B). We observed significant cleavage as measured by substrate fluorescence when using the active (on-target) gRNA, compared to the control (off-target) gRNA, showing Cas9 guide-mediated specificity is maintained. Notably, we observed significantly more cleavage without the lipid coating than with, supporting our earlier conclusion that the lipid-coating protects the loaded Cas9 from the external environment. Second, an endolytic protease, proteinase-K, was used in a protection/degradation assay (Figs. 3C, S5). Cas9 is a monomeric protein, approximately 1380 amino acids in length, with 689 predicted proteinase-K cleavage sites^34^. Proteinase-K rapidly degraded free RNP in solution and RNP bound to MSNs. However, the lipid coating (LCMSN samples) offered partial protection against degradation of the Cas9, suggesting that some fraction of RNP is inaccessible to external enzymes in the coated particles.

### Section 3: LCMSNs successfully deliver Cas9 RNP cargo intracellularly with minimal cell toxicity

**Figure 4:**
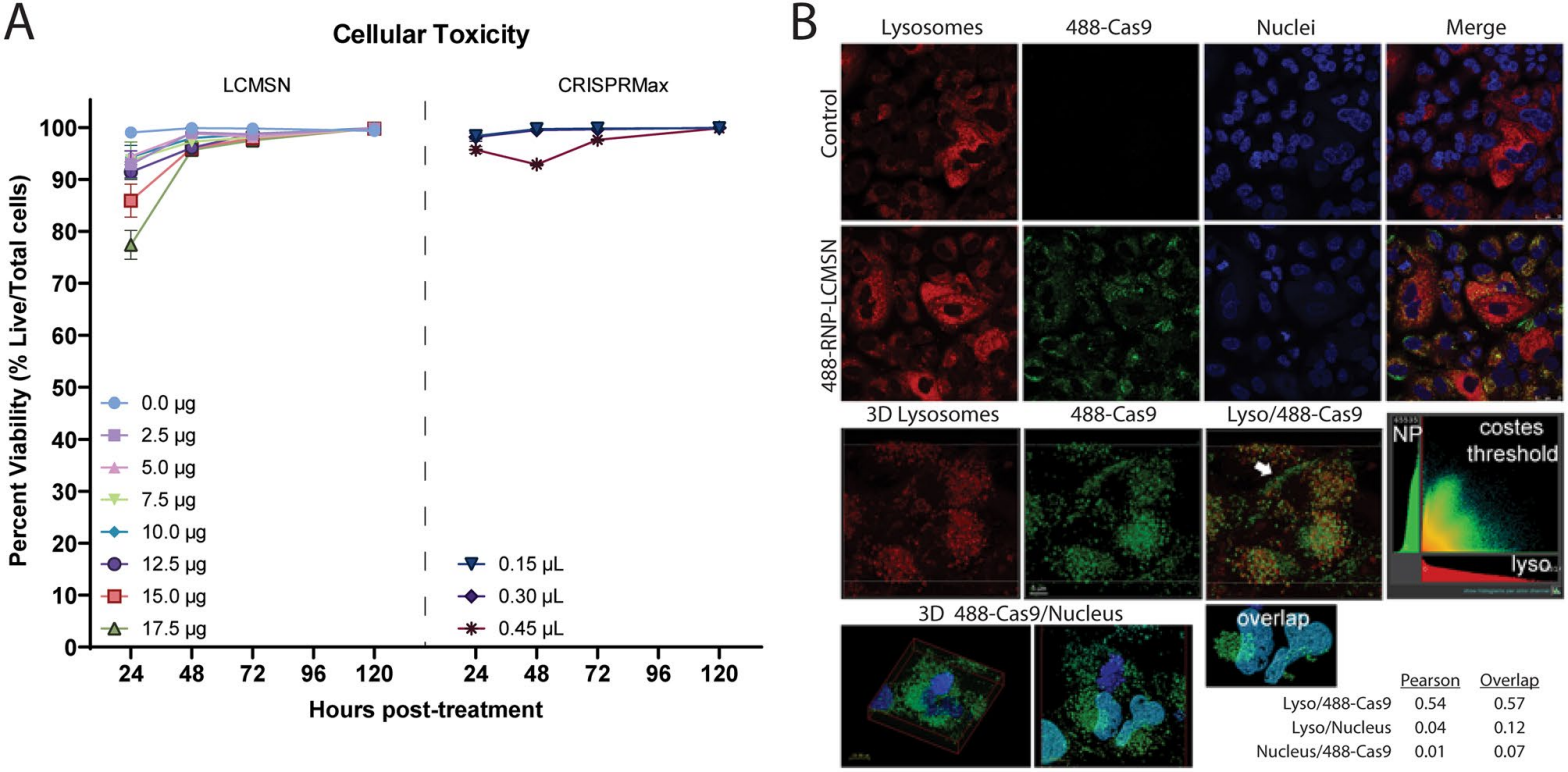
LCMSN cellular delivery occurs via an endosomal pathway with limited cytotoxicity. (**A**) Toxicity of LCMSNs was compared to CRISPRMax in A549 cells at 24, 48, 72, and 120 h post-treatment. (**B**) Confocal microscopy was performed on cells 24 h post-treatment with Alexafluor 488-tagged RNP LCMSN. Colocalization measurements (Pearson/overlap) between various markers are indicated (lyso = lysosome; NP = nanoparticle).

To determine toxicity of the LCMSNs we performed a cellular toxicity assay (Fig. 4A). CRISPRMax lipofection reagent was used as a control to represent toxicity associated with conventional CRISPR RNP delivery reagents. For this assay, percentage of dead cells was determined using a dead (propidium iodide)/total (Hoechst) counterstaining protocol, with cells assessed at 24, 48, 72, and 120 h post-treatment with either LCMSNs or CRISPRmax. Although we saw some initial toxicity 24 h post-treatment with high dosages of both LCMSN and CRISPRmax, the LCMSN formulations did not show substantial cell toxicity at later time points, even at high dosage levels.

To understand the intracellular trafficking of the RNP when delivered to cells via LCMSNs, Alexa Fluor 488-conjugated Cas9 RNPs were loaded into LCMSNs (488-RNP-LCMSNs) and used to treat A549 cells. At 24 h, the 488-RNP-LCMSN treated cells, along with untreated control cells were fixed and imaged using confocal microscopy. As previous studies suggest that RNP-LCMSNs are internalized through clathrin-mediated endocytosis^23^, here we explored the extent at which RNP-LCMSNs traffic to lysosomes. The basic function of lysosomes is to aid in the degradation of proteins and lipids and therefore limited nanoparticle trafficking to these cellular organelles is preferred. Colocalization analysis indicated that 57% of the 488-Cas-RNP overlapped with a lysosome stain, and 7% overlapped with a nuclear stain (Fig. 4B). These results suggest that although a majority of the RNP was associated with lysosomes, a fraction of the RNP escaped or avoided lysosome trafficking and was available to localize to the nucleus for genome editing activity.

### Section 4: Cas9 RNP delivery by LCMSNs successfully target host-genes essential for viral infection in vitro and edits a reporter gene in vivo

**Figure 5:**
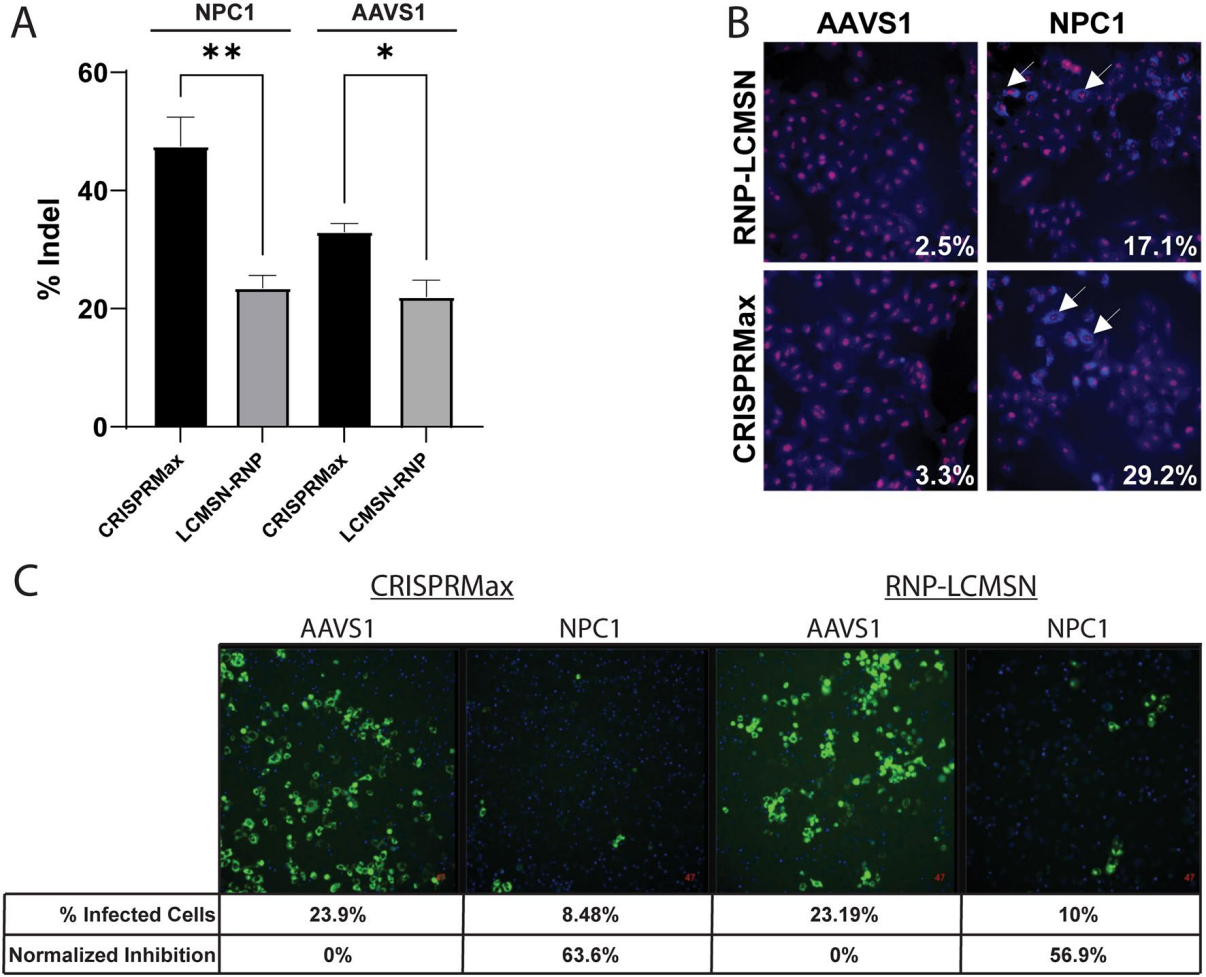
RNP-LCMSNs prevent viral infection in vitro. (**A**) Percent of Insertion/Deletion (Indel) mutations following treatment with RNPs targeting NPC1 or AAVS1 genes in A549 cells as determined by Synthego ICE analysis. **p < 0.005; *p < 0.05. (**B**) Filipin staining of edited cells showing accumulation of free cholesterol following NPC1 knock-out (KO). Arrows indicate filipin staining examples and cells were counterstained with propidium iodide (red). (**C**) Percent of cells infected in NPC1 KO and AAVS1 KO A549 cell populations following inoculation with VSV-EBOV-GFP (MOI = 0.02).

To demonstrate efficacy in vitro, we chose to target NPC1, the known entry receptor for EBOV, using RNP-LCMSNs, along with the adeno-associated virus integration site 1 (AAVS1), to serve as a CRISPR target with no impact on viral infection. While CRISPRmax resulted in a higher percent of InDels than LCMSN delivery of RNPs, the LCMSN delivery resulted in approximately 20% editing against both targets (Fig. 5A). Knockout of the cholesterol transporter function of NPC1 results in intracellular cholesterol accumulation that can be visualized through filipin staining. NPC1 targeted RNP-LCMSNs resulted in a greater accumulation of filipin (17.1% of cells) compared to targeting of the AAVS1 control (2.5%) (Fig. 5B). Although CRISPRmax resulted in higher levels of filipin staining (29.2%), the LCMSN delivery was still effective. Next, we performed cell infection assays with a VSV-EBOV-GFP pseudotyped virus at 0.02 and 0.1 MOI in cells treated with CRISPRmax or RNP-LCMSNs with gRNAs targeting either AAVS1 or NPC1 (Fig. 5C, Video S1). RNP-LCMSN mediated knockdown of NPC1 significantly inhibited VSV-EBOV-GFP infection as the percent of GFP-positive cells was reduced by 56.9% at 48 h post infection compared to AAVS1 targeting.

**Figure 6:**
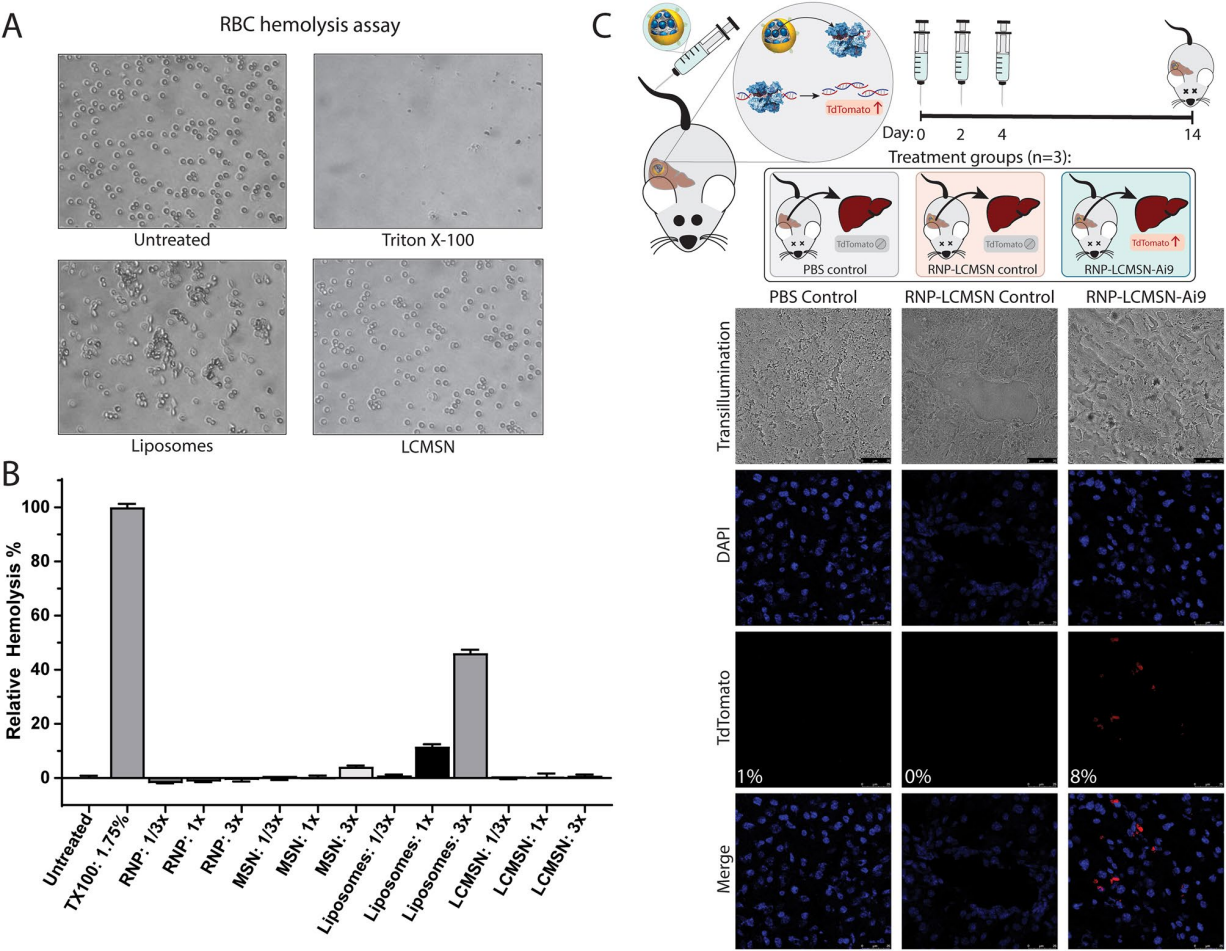
RNP-LCMSN in vivo delivery results in successful editing of the mouse liver. (**A**) Representative images of RBCs following a hemolysis assay performed at pH7.4 under indicated treatment conditions. (**B**) Percent of RBCs that underwent hemolysis following treatment relative to 1.75% Triton X-100. 1x = equivalent dosage to the 1 mg/mouse dose. (**C**) Ai9-tdTomato reporter mice were treated with RNP-LCMSNs targeting the tdTomato stop cassette (RNP-LCMSN-Ai9) or control sequence (RNP-LCMSN-control). Representative images from each treatment group are shown. Additional tissue sections are shown in Fig. S7.

LCMSN and other lipid-based delivery methods have historically experienced concerns surrounding hemolytic effects following vascular delivery. Red blood cell (RBC) hemolysis assays showed that at a pH of 7.4 the LCMSN particles did not induce lysis, while Triton X-100 did (Fig. 6A, B). Interestingly, DCD3330 liposomes at high concentration resulted in RBC lysis (Fig. 6B). However, the degree of lysis seen with the LCMSN particles remained minimal, even at 3 × the standard treatment concentration.

Importantly, to demonstrate LCMSN cargo delivery in vivo, Ai9-tdTomato reporter mice were treated with RNP-LCMSNs. Previously, in vivo delivery of RNP-LCMSNs resulted in 10% editing in Ai9 reporter mice via localized intracranial injection^23^; however, systemic delivery with those nanoparticles was not assessed. Here, the optimized RNP-LCMSNs were first tested using localized intramuscular delivery to ensure their initial in vivo efficacy and relative safety (Fig. S6). After achieving intramuscular delivery, Ai9-tdTomato reporter mice were treated with 3 doses at 1 mg per dose of LCMSNs suspended in 200 µL PBS via systemic administration. The RNP-LCMSN formulations resulted in 8% expression of the tdTomato reporter in liver sections, whereas the control scramble RNP LCMSN treated mice showed no reporter expression (Fig. 6C). Importantly, no reporter activity was seen in other tissues, suggesting that off-target effects were minimal (Fig. S7). While 8% tdTomato liver expression via treatment with RNP-LCMSNs is similar to liver editing levels of RNP delivered by LNPs, in vivo protection against a surrogate EBOV challenge would likely require greater than 50% editing of the host Npc1 gene target. We therefore did not proceed to perform in vivo anti-viral efficacy studies^24^. Overall, by successfully targeting endogenous genes in vitro and systemically delivering CRISPR RNP in vivo, we extend the utility of the LCMSN platform for gene editing applications.

## Conclusions

In conclusion, LCMSN can be used for successful delivery of RNP for CRISPR-Cas9-mediated editing of host genes to prevent viral infection. Here, we have found that the unmodified stellate nanoparticle offered the best delivery vehicle when partnered with our DCD3330 lipid-coating formulation. Furthermore, this LCMSN successfully knocked-down NPC1 expression in vitro, resulting in impaired viral infection of VSV-EBOV, a high-pandemic risk pathogen surrogate. DCD3330 RNP-LCMSN also showed successful editing in vivo in a reporter mouse model, with editing limited to liver tissue. This delivery formulation has potential therapeutic use targeting host genes necessary for EBOV infection and other pathogens where the liver is a predominant disease target. However, further work will be necessary to optimize the CRISPR-based editing systems used for therapeutic approaches, to both ensure safety and achieve editing rates necessary for a range of applications. Newer CRISPR technologies, such as CRISPRinhibition^35^ and CRISPRoff^36^, may provide promising avenues to address these concerns by silencing genes without risks of DNA mutation.

## Materials and methods

All methods were performed in accordance with institutional guidelines and regulations.

### CRISPR-Cas9 protein and guide RNA reagents

All Cas9 proteins used in this study were derived from the prototypic *Streptococcus pyogenes* species. Cas9 protein was produced as described in Seamon et al.^33^ and contained a N-terminal nuclear localization signal and a C-terminal HIS tag. For in vivo studies, endotoxin-free Cas9 was purchased from IDT (IDT, 1081058). For microscopy experiments using labelled Cas9, the AlexaFluor 488 protein-labeling kit was used per manufacturer protocols (ThermoFisher, A10235). Re-purified Cas9-488 was assayed to confirm enzymatic activity in vitro. In vitro transcription of gRNA was performed according to https://www.protocols.io/view/In-vitro-transcription-of-guide-RNAs-dwr7d5, using Phusion Flash Polymerase (ThermoScientific), TranscriptAid T7 Kit, and MEGAClear Transcription Clean-Up Kits (Thermo Scientific). For the Stoplight/AAVS1 targeting-gRNA, the guide sequence was 5′-GGGGCCACTAGGGACAGGAT-3′. For targeting the Ai9-Reporter locus, end-modified guide was purchased from Synthego using the guide sequence of 5′-AAGTAAAACCTCTACAAATG-3′ reported in Staahl et al.^37^*.* The NPC1 guide-target was chosen by empirically screening 4 Cas9 candidate guides with targets in Exons 6 and 8 for efficacy, with the best guide being pursued. The selected guide (hNPC1-Exon8-gRNA1) for LCMSN studies was purchased from Synthego, with the sequence 5′-AGTATCTGTATGTCAAGCGG-3′.

### Fabrication and characterization of silica nanoparticles (Plain and MSNs)

Stӧber nanoparticles and mesoporous silica nanoparticles of different shapes and pore structures were generated as previously described in the literature^17,19,27,38–40^. MSNs were made via a hydrolytic sol gel method under base catalytic conditions and using cationic surfactant as structure directing agent and porogen, and TEOS (Sigma-Aldrich) as silica precursor. The specific procedures for synthesis of bare MSNs, as well as functionalization of stellate MSNs by polyethylene glycol (PEG) and nickel charged nitrilotriacetic acid (NiNTA), are described in the supporting information (SI). Upon washing and surfactant extraction, all resulting MSNs were resuspended in pure ethanol and passed through a 1 µm glass filter (Millipore-Sigma) to remove any residual aggregates. Size and zeta potential were assessed using a Zetasizer instrument (Malvern Instruments, Ltd.) and morphology was assessed by TEM (JEOL 2010).

### Preparation of DCD3330 liposomes

The DCD3330 liposome suspension in 0.5xPBS (supplemented with 4 mM MgCl2) composed of 33 mol% 1,2-dioleoyl-3-trimethylammonium-propane (DOTAP), 33 mol% 1,2-dioleoyl-sn-glycero-3-phosphoethanolamine (DOPE), 30 mol% Cholesterol, and 4 mol% 1,2-distearoyl-sn-glycero-3-phosphoethanolamine-N-[carboxy-(polyethylene glycol)-2000] (DSPE-PEG2000) (Avanti Polar Lipids) was prepared at 15 μmol/mL, which corresponds to 7.2 mg/mL. Stock lipids were prepared in chloroform (Sigma) and were combined in a glass scintillation vial then dried down using a rotary evaporator (Buchi Corp.). The resulting lipid films were placed under vacuum overnight to ensure complete solvent removal then resuspended in 1 mL 0.5 × PBS 4 mM MgCl_2_ solution. The suspension was placed in a sonication bath (Branson) and sonicated for 30 min at 30 °C, then immediately extruded with 21 passes through a 100 nm extruder and filter set (Avanti Polar Lipids). Hydrodynamic size and charge of liposomes were assessed by a Malvern Zetasizer.

### LCMSN fabrication, RNP loading, and characterization

MSNs were spun down in a microcentrifuge at 21,000×*g* for 10 min to remove ethanol, and washed with 1 mL of water in a sonicating water bath. Washed MSNs were collected, centrifuged, and resuspended into water at 10 mg/ml concentration. CRISPR RNP was precomplexed at a 5:1 ratio by weight of Cas9:gRNA at 0.375 mg/ml in loading buffer (100 mM NaCl, 50 mM Tris, 10% glycerol, pH 8.0) at room temperature. RNP was loaded by addition of RNP-solution at a 1:5 Cas9:MSN ratio by weight, during active pipetting and brief sonication (5 s), and then incubated in a static heat-block at 25 °C for 30 min. The freshly prepared DCD3330 liposomes were then fused to RNP-loaded MSN by addition of the prepared liposomes at 7.2:1 liposomes:MSN ratio by weight, during active pipetting and sonication in a water bath. The resulting LCMSNs were collected by centrifugation at 15,000×*g* and washed with 1 mL PBS before aspiration. The RNP-LCMSN pellet was then resuspended in PBS at a concentration of 1 mg/mL (MSN weight), by pipetting and gentle sonication. RNP-LCMSNs were then assessed for size and dispersity by DLS (Malvern Zetasizer).

### Protection and release of RNP loaded LCMSNs

LCMSNs (30 μg) were suspended in PBS and collected into 1.5 mL microcentrifuge tubes and supplemented with either PBS or PBS-containing 1.8% triton X-100 detergent to destabilize the lipid-coating. Tubes were incubated on an Eppendorf Thermostat, set at 37 °C with shaking at 400 rpm, for 4 h. Tubes were then centrifuged at 21,000×*g* for 10 min to separate the supernatant from the remaining MSNs. Laemmli-sample buffer (BioRad) was added, and samples were denatured at 98 °C for 10 min prior to loading onto 10% acrylamide SDS-PAGE gels (Biorad) and imaged after incubation with Coomassie blue protein staining solution (ThermoScientific). Loading and release was assessed by densitometry (ImageJ, ProteinSimple Imaging).

### Regular and cryogenic electron microscopy

MSNs were diluted to 100 μg/mL and 10 µL were dropped on to copper grids and maintained at ambient temperature for 10 min to allow for samples to dry. Grids were imaged at 200 keV using a DE-20 (Direct Detector Inc.) direct electron detector camera. The energy selecting slit was set to 20 eV and the microscope had a field emission electron source and omega-type electron energy filter to remove inelastically scattered electrons from the image formation. A DE-20 camera was used to collect images in movie mode with a frame rate of 25 frames/second. After image collection, frame alignment was performed using the DE_process_frames.py script provided by Direct Electron Inc. Images were collected at 40,000× magnification and the pixel size on the specimen scale corresponded to 1.5 Å/pixel. For cryo-electron microscopy analysis, LCMSNs were prepared as above and vitrified using an automatic plunge freezer (Leica). LCMSN solution was added to a C-flat grid (Protochips, Inc) with 2 μm holes and blotted with filter paper. Grid was flash frozen in liquid ethane and stored in liquid nitrogen until being transferred to a JEM 2200FS electron microscope (JEOL) and imaged as described above.

### Generation of the CRISPR reporter cell line

The stoplight CRISPR reporter system was generated in A549 cells, using a fluorescent reporter system construct cloned by PNA Bio (Fig. S1). Parental A549 cells were obtained from the American Type Culture Collection (ATCC). The CRISPR reporter construct was packaged and integrated using a lentiviral delivery system. Lenti-transduced cells underwent antibiotic selection for approximately one week prior to utilization of a BD FacsMelody sorter, which was used to establish clonal lines positive for constitutive expression of red fluorescence. Clones were then screened and validated by transfection of RNP using commercial reagent. The chosen clone was then expanded for use in all studies and maintained in F-12K media (Gibco) supplemented with 10% FBS and 1% penicillin/streptomycin.

### CRISPR cell reporter genome editing assays

In brief, cells were seeded into tissue-culture plates in F-12K media supplemented with 10% FBS and 1% penicillin/streptomycin (Gibco) 24 h prior to LCMSN treatments. Editing experiments were typically performed in 12-well plates (Costar) with a seeding density of 50,000 cells/well. LCMSNs in PBS at a concentration of 1 mg/ml were added directly to cells from 20 to 80 μg/well, and the amount of RNP used to treat cells was calculated at 140 µg per 1 mg of MSN. Control cells were transfected using RNP loaded into DCD3330 liposomes. Following overnight incubation, cells were washed, and fresh media was added. After 72 or 96 h of expansion, cells were assayed as described per experiment. For flow cytometry analysis of CRISPR reporter editing, wells were washed with PBS, and cells were detached via trypsin treatment. Cells were collected, centrifuged to remove residual trypsin, and resuspended in PBS supplemented with 2% FBS and 1% paraformaldehyde. Fixed samples were run on an auto-sampler-capable BD Accuri C3, with filtered gating on FSC and SSC for cell-size, and analyzed for percent of GFP+ cells. CRISPRMax treated off- and on-target RNP guides were used as negative and positive controls respectively for gating.

### RNP protection and activity assays when formulated in LCMSNs

Proteinase-K (ThermoFisher) was used to assess the relative amount of accessible RNP. Briefly, tubes containing either free RNP, RNP bound onto MSNs, or RNP encapsulated into LCMSNs were incubated with Proteinase K and incubated at 37 °C in PBS. Samples were taken at both 1 and 15 min timepoints and placed in 99 °C pre-heated Laemmli sample buffer (BioRad) to denature and stop the reaction. Samples were then analyzed via Coomassie-stained SDS-PAGE gels.

RNP activity on particles was determined via a fluorescence resonance energy transfer (FRET) assay modified from Seamon et al.^33^. Briefly, the FRET-based assay uses a fluorophore/quencher-labeled DNA substrate which increases in fluorescence upon Cas9 cleavage. RNP using either FRET-targeting gRNA or non-targeting gRNA were loaded onto MSNs, and either assayed directly or lipid-coated as described above to generate LCMSNs. Samples were incubated with fluorescent substrate for 1 h, and the reactions were quenched as described. Samples were read on a Tecan plate reader.

### Cellular toxicity assays

A549 cells (ATCC) were seeded at confluency into 96-well optical-grade tissue culture plates (Corning Scientific), and treated with either equal volumes of a dosage series of LCMSN or transfected using CRISPRMax, as described above. After 16 h, the media was replaced. Technical replicates were analyzed separately at 24-, 48-, 72-, and 120-h post-treatment. Fifteen minutes prior to the timepoint, cells were incubated with a live/dead cell staining mixture comprised of Hoechst and propidium iodide (Fisher Scientific), washed with PBS, and then overlaid with LiveCell Imaging Media (Fisher Scientific). A CX7 high-content imager (Thermo Scientific) was used to automate acquisition of 10 fields of view per well at the indicated timepoints. Percentage of dead cells was calculated by the CX7 software, utilizing a protocol in which cells positive for propidium iodide staining were counted against the total cell count (Hoechst-staining). Values for each well were averaged and plotted in GraphPad PRISM.

### Confocal microscopy

Confocal microscopy was performed as described previously^23^. Briefly, A549 cells (ATCC) were grown on coverslips in 6-well plates overnight at 50,000 cells/well. Following overnight adhesion of cells to coverslips, cells were treated with 25 μg LCMSN loaded with a Cas9-RNP-488 and incubated for 24 h, with labeling as described under CRISPR protein and nucleic acid reagents above. 50 nM LysoTracker Red was added to the culture for the final 45 min, and then, cells were washed with PBS and fixed using 4% paraformaldehyde for 1 h. Cells were counterstained with DAPI in the mounting media (Prolong) to visualize nuclei.

### Filipin staining

Filipin Complex III (Sigma) was used to image free cholesterol in NPC1-targeted samples. In brief, LCMSN-treated cells were fixed with 3% paraformaldehyde for 2 h at room temperature. PBS with 1.5 mg/ml glycine was used to quench, and following PBS rinses, filipin was used at 0.05 mg/ml in a PBS solution supplemented with 10% fetal bovine serum. After a 3-h incubation, cells were rinsed and incubated with propidium iodide supplemented PBS for 15 min. Cells were rinsed again prior to microscopic imaging, which was done using filter sets with excitation/emission of 386 nm/440 nm and 549 nm/600 nm for filipin and propidium iodide, respectively. Images were auto-acquired using a CX7 High-Content Imager (ThermoScientific).

### Genomic DNA extraction and editing analysis

Genomic DNA was harvested from treated cells using commercially available extraction kits (Epoch Sciences). PCR was performed using the primers listed in Table 1, flanking the edited sites. PCR products were column purified using commercially available kits (Epoch Sciences) and sent for Sanger sequencing (Genewiz Inc) using the primers listed below. Chromatograms were uploaded into Synthego’s ICE sequence decomposition software to obtain relative ICE editing (% of InDels) as well as knockout scores (% InDels resulting in frameshifts).

**Table 1:** Primers for amplification and sequencing of endogenous editing.

Gene	PCR forward primer	PCR reverse primer	Sequencing primer
NPC1	CGCCACTGAAGTCTTCTTTCTC	GGTCTCATATGTGTCCCCAGG	ATATACCATGACATTCAGCCCC
AAVS1	TGGACAACCCCAAAGTACCC	ACCAGGATCAGTGAAACGCA	CTCTGGCTCCATCGTAAGCA

### Viral entry assays

A549 cells (ATCC) were treated with LCMSN or CRISPRMax as described for each assay. After 3 days of treatment, cells were lifted and plated into multiple new tissue culture plates, allowing for assay replicates from the parental LCMSN treated wells. Virus infection assays were performed using VSV-EBOV-GFP, a replication-competent pseudovirus expressing green fluorescent protein known to mimic authentic EBOV cell entry. Cell infections with VSV-EBOV-GFP were performed at a multiplicity of infection (MOI) = 0.02 and 0.1 as indicated. For microscopy and time course analysis, cells with 100 μl of media had up to 20 μl of virus-diluted in media overlaid on top. Eight hours after infection, an overlay of DAPI-staining media was added, and the plates were washed and overlaid with fresh media. A CX7 high-content imager was used, starting at 10 h post infection, with a built-in incubation chamber holding normal tissue culture temperature, CO_2_, and humidity levels. A kinetic analysis protocol was run to image multiple fields per well of the plate every hour for 48 h, in the DAPI and GFP channels. The CX7 analysis automatically generated the time-course videos, which were overlaid together using Adobe Premiere. For flow cytometry analysis, LCMSN or CRISPRMax treated cells were plated in 12-well tissue culture plates (Corning) and infected as above with VSV-EBOV-GFP. 48 h after infection, the cells were washed in PBS, trypsinized, and fixed in 4% ethanol before being resuspended into a flow-cytometry buffer of sterile saline supplemented with 1% fetal bovine serum. Samples were run on an auto-sampler-capable BD Accuri C3, with filtered gating on FSC and SSC for cell-size, and analyzed for percent GFP+ cells utilizing an uninfected cell sample as a thresholding control.

### Hemolysis assay

Erythrocytes from CD-1 ICR mice were purchased from BioIVT. The pH responsive nanoparticle hemolysis assay was performed as based on Evans et al.^41^, using either loaded LCMSN or the individual constituents incubated with the erythrocytes in PBS. The assay was performed at pH 7.4 to represent cytosolic conditions and at pH 5.6 to represent late endosomal conditions. The LCMSN:Red Blood Cell (RBC) ratios utilized represent physiologically relevant concentrations proportional to 1 mg of LCMSN treatment delivered systemically per mouse. Assay parameters were calculated using representative values, obtained from Jackson Laboratory, of average blood volume (72 mL blood/kg of body weight) and hematocrit percentage per mouse (52%) to achieve this ratio ex vivo. Absorbance values were read on a Tecan Spark, normalized to controls, and plotted using GraphPad Prism.

### In vivo studies

All animal work was conducted in accordance with protocols approved by the University of New Mexico (UNM) Institution Animal Care and Use Committee and are reported in accordance with the ARRIVE guidelines. Ai9-tdTomato reporter mice were purchased from Jackson Laboratory (007909) and all experiments were performed with 6- to 10-week-old mice. Briefly, a tdTomato reporter with a loxP-flanked STOP cassette is inserted into the Gt(ROSA)26sor locus such that the STOP cassette prevents CAG promoter driven expression of the reporter unless Cre-mediated recombination occurs. RNP-LCMSNs were prepared as described above using either RNP complexed with on-target gRNA (Ai9-gRNA) or a scrambled gRNA control. Three mice per group (RNP-LCMSN-Ai9; RNP-LCMSN-control) received 1 mg (LCMSN weight) per dose in 200 µl total volume diluted in PBS, with three total doses administered per mouse on days 0, 2 and 4 via tail vein injection. Similarly, a set of control mice (n = 3) were given PBS following the same dosing schedule. Animals were sacrificed 14 days after the initial treatment and tissues were harvested. Tissues (liver, spleen, lungs, kidneys, brain) were snap frozen at dissection and sent for frozen sectioning. Sectioned tissues were mounted using ProLong Gold containing DAPI nuclei stain and imaged on a Lecia SP8 confocal microscope. Images were used to calculate the percentage of cells expressing tdTomato. Adobe Photoshop was used to apply pseudocolor to the grayscale images. The guide for tdTomato was used as from Staahl et al.^37^, listed above.

## Supplementary Information

Supplementary Video 1.

Supplementary Information 1.

## Data Availability

The datasets generated and analyzed during the current study are available from the corresponding author upon reasonable request.

## Abbreviations

CRISPR: Clustered regularly interspaced short palindromic repeats
LCMSN: Lipid-coated mesoporous silica nanoparticles
RNP: Ribonucleoprotein
NPC1: Niemann–Pick disease type C1
EBOV: Ebola virus
SARS-CoV-2: Severe acute respiratory syndrome Coronavirus 2
gRNA: Guide RNA
GFP: Green fluorescent protein
InDel: Insertion/deletion mutation
PEI: Polyethylenimine
eGFP: Enhanced green fluorescent protein
VSV: Vesicular stomatitis virus
PEG: Polyethylene glycol
NiNTA: Nitrilotriacetic acid
SI: Supporting information
TEM: Transmission electron microscopy
CryoEM: Cryogenic electron microscopy
DOTAP: Dioleoyl-3-trimethylammonium propane
DOPE: Dioleoylphosphatidylethanolamine
DSPE-PEG2000: 1,2-Distearoyl-sn-glycero-3-phosphoethanolamine-N-[carboxy-(polyethylene glycol)-2000]
DLS: Dynamic light scattering
PBS: Phosphate buffered saline
rpm: Revolutions per minute
SDS-PAGE: Sodium dodecyl-sulfate polyacrylamide gel electrophoresis
FBS: Fetal bovine serum
FSC: Forward scatter
SSC: Side scatter
FRET: Fluorescence resonance energy transfer
RBC: Red blood cell
RFP: Red fluorescent protein
MOI: Multiplicity of infection
AAVS1: Adeno-associated virus integration site 1
KO: Knock-out
