# Supplementary material for: Lipid-coated mesoporous silica nanoparticles for anti-viral applications via delivery of CRISPR-Cas9 ribonucleoproteins
Source: Sci Rep. 2023 Apr 27;13:6873. doi: 10.1038/s41598-023-33092-4 (PMC10133914; doi:10.1038/s41598-023-33092-4)
Supplement: Supplementary file 2 — Supplementary Information 1. [file 41598_2023_33092_MOESM2_ESM.pdf]

Lipid-coated mesoporous silica nanoparticles for anti-viral applications via delivery of CRISPR-Cas9 ribonucleoproteins

Authors: Annette E. LaBauve[1#], Edwin A. Saada[2,3#], Iris K. A. Jones[2], Richard Mosesso[2], Achraf Noureddine[4,5], Jessica Techel[1], Andrew Gomez[6], Nicole Collette[3], Michael B. Sherman[7], Rita E. Serda[8], Kimberly S. Butler[9], C. Jeffery Brinker[4,5,10], Joseph S. Schoeniger[2], Darryl Sasaki[1], and Oscar A. Negrete[2,]*

SUPPLEMENTAL MATERIALS & METHODS

Stober

In a 100 mL round bottom flask, add TEOS (1.5 mL) to a solution of ammonia (28%, 2 mL) to ethanol (95%, 40 mL) heated to 40˚C. Keep stirring at 600 rpm for 6 hr to form silica nanospheres of about 200 nm diameter. Centrifuge the suspension at 20,000 rcf for 20 min and resuspend the isolated pellet in ethanol. Wash two more times following the same centrifugation/suspension steps and store the suspension at 4˚C.

MSN Hex

MSN Hex particles were formulated as described previously [16, 31]. Briefly, hexagonally shaped MSN were prepared by dissolving CTAB (290 mg) in 150 ml of aqueous ammonium hydroxide

(0.32 M) in a 250 mL beaker. The reaction was covered with parafilm (Bemis) and heated to 50°C in a silicone oil bath for 1 hr stirring at high speed (650 rpm). A TEOS solution was prepared at 0.88 M in ethanol (3 mL) and combined with the Cy3-APTES solution, then added to the CTAB solution and stirred vigorously at 50°C for one hour, unsealed then aged overnight (~18 h) in the 50°C oil bath without stirring. The remaining volume was then transferred to a 100 mL glass bottle and capped for an overnight hydrothermal treatment at 70°C. The MSN suspension was then centrifuged at 50,000 rcf for 15 minutes and particles were washed twice with 100% ethanol. CTAB removal was achieved by resuspending particles in 20 mL of 6 g/L ammonium nitrate in ethanol and sonicating at 40°C for 20 min. Particles were collected by centrifugation, washed with 95% ethanol, collected by centrifugation, resuspended in 20 mL of an ethanolic HCl solution (1%) and sonicated twice for 20 min at 40°C. Particles were washed with 90% ethanol followed by 100% ethanol, collected by centrifugation, and resuspended in 15 mL of 100% ethanol.

MSN dendritic

In a 100 mL round bottom flask, 0.18 g (1.8 mmol) TEA, 24 mL (72.6 mmol) CTAC, and 36 mL of distilled water were stirred at 400 rpm and heated to 50°C for an hour to bring the solution to temperature. the stirring speed was adjusted to 250 rpm before 20 mL of a solution of TEOS in cyclohexane (10% v/v) was added to form the biphasic system. The reaction was kept at 50°C and under stirring for 16 h. The mixture was then kept without stirring for 15 min and the top organic phase was removed (as much as possible). The bottom aqueous phase containing the nanoparticle suspension was centrifuged and the isolated pellet washed in ethanol through successive sonication centrifugation steps. The removal of CTAC was achieved by washing the suspended particles in NH_4NO_3 (6 g/L) in ethanol followed by 1% HCl in ethanol. Each suspension was sonicated for 15

minutes and centrifuged accordingly. Each cycle of centrifugation was performed at 50,000 rcf for 15 minutes at room temperature. All MSN samples were fully characterized and stored in pure ethanol.

MSN stellate

In a 50 mL round bottom flask, TEA (70 mg), CTAT (0.3 g) and water (20 mL) were mixed at 80°C for 30 min. The stirring speed was adjusted to 1250 rpm and TEOS (2 mL) was added and the condensation was kept ongoing for 2 hr. The mixture was then cooled and spun down. Surfactant removal was achieved by successive washing steps by NH_4NO_3 (6 g/L ethanol) and HCl (1% ethanol, twice); each step included 15 min sonication and centrifugation. All centrifugation cycles were done at 50,000 rcf for 10 min at 20°C.

Stellate PEG

Bare stellate MSN (25 mg) were suspended in 190 proof ethanol at 5 mg/mL. A solution of PEG-silane 2000 (NANOCS, PG1-SL-2k) 10 mg/mL in 190 proof ethanol (1 mL) is added to the MSN suspension followed by 2 μL of triethylamine. The reaction is kept overnight agitating at room temperature. The suspension is then centrifuged, and the isolated pellet is washed twice with 190 proof ethanol and stored in 200 proof ethanol. PEG functionalization was monitored by zeta potential and the charge increased from -28 mV to -13 mV suggesting that neutral PEG had been added to the silanol surface.

Stellate Ni-NTA

Aminated MSN (10 mg) were suspended in DMSO and incubated with dithiobis(succinimidyl propionate, DTSP, 30 mg) overnight. After centrifugation (20 min, 20,000 rcf) a solution of NTA (20 mg) in DMSO (4 mL) was added and reacted with the free NHS from DTSP for 24hr. The NTA-MSN was washed in water and a solution of nickel sulfate (1M, 2 mL) was added to form the Ni-NTA MSN. After washing with water, the particles were considered ready for loading with a HisTag RNP. All functionalization steps were monitored by zeta potential (Figure S8).

In vivo study

Animal work was conducted in accordance with protocols approved by the Lawrence Livermore National Laboratory (LLNL) Institution Animal Care and Use Committee. Homozygous *tdTomato* mice, as described previously, were used for this experiment. (The Jackson Laboratory, Farmington, CT, Cat# 007909). RNP-LCMSN were formulated as for previous experiments to target the tdTomato cassette. These nanoparticles were injected into gastrocnemius muscles (20 µl per muscle) using a 35 g WPI NanoFil syringe. Two weeks after the injection, the muscles were harvested, fixed on the bone in 10% neutral-buffered formalin, and then dissected and processed for paraffin histology using a Leica ASP300S tissue processor (Buffalo Grove, IL). Six-micron sections were cut using a rotary microtome. After dewaxing, antigen retrieval was done at 65°C for 30 minutes using Uni-Trieve (Innovex, Cat# NB325). Slides were blocked using Rodent Block M (BioCare Medical RBM 961L). Slides were stained with a rabbit polyclonal antibody against Red Fluorescent Protein (RFP) at 1:400 in 5% BSA with 0.1% Triton X-100 (Rockland, cat# 600-401-379) at room temperature followed by incubation for 2 hr with highly cross-adsorbed goat anti-rabbit secondary Alexa Fluor 594 antibody at 1:1000 (Invitrogen, Cat# A-11037) and mounted

using Prolong GOLD with DAPI (Invitrogen, cat# P36931). Stained slides were imaged using a Leica DMi8 microscope at 400 x magnification.

SUPPLEMENTAL FIGURES

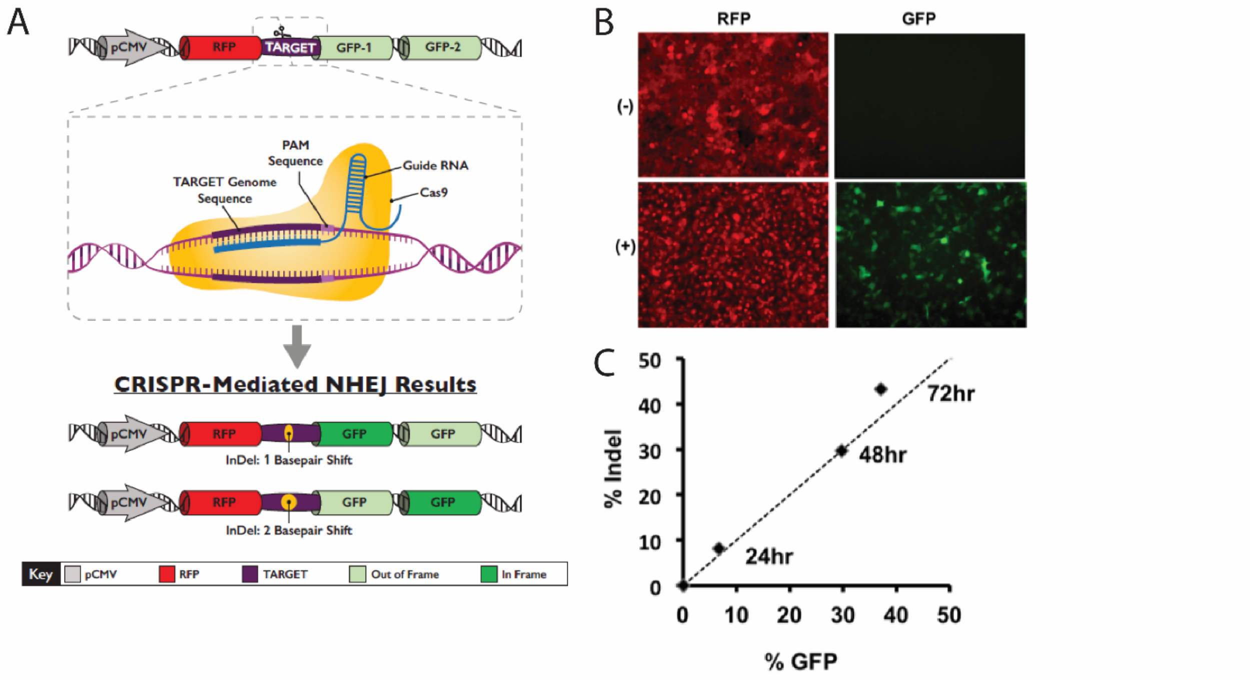

Figure S1. RFP/GFP CRISPR editing reporter assay. (A) A549 cells in which RFP and GFP are linked through an Cas9 endonuclease targeting sequence enable quantification editing by measuring GFP production in cells constitutively expressing RFP. (B) Example of successful GFP reporter expression following CRISPR-mediated editing. (C) Percent of InDels versus percent GFP+ cells at 24, 48, and 72 hours post-treatment with DCD3330 RNP-LCMSNs.

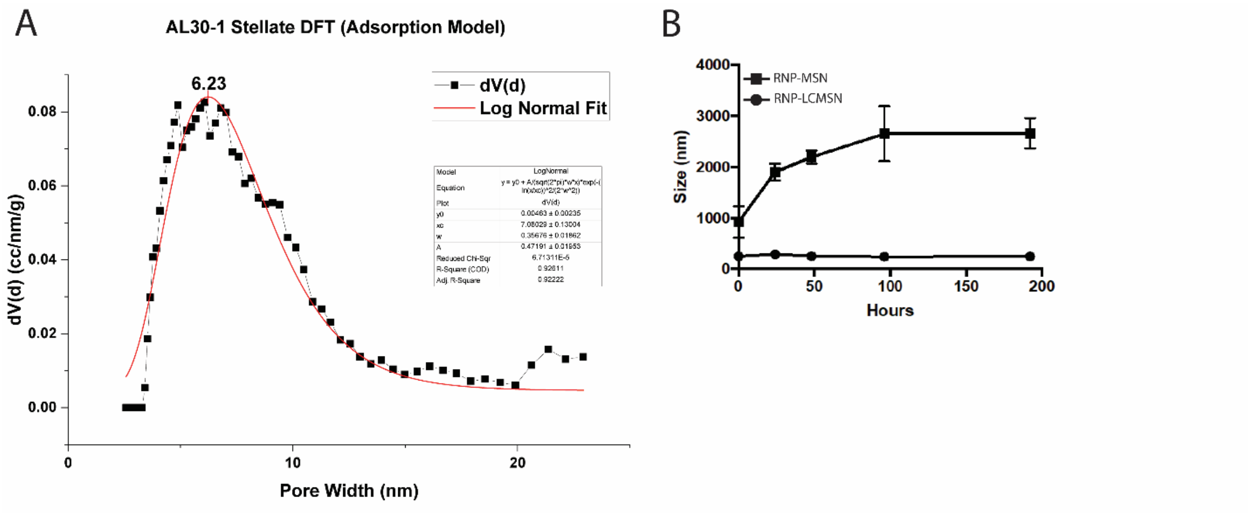

Figure S2. Characterization of pore size and stability of stellate MSNs. (A) Porosimetry of unmodified stellate MSN. (B) Size of particles (nm) for RNP-loaded MSN and RNP-loaded LCMSN over time.

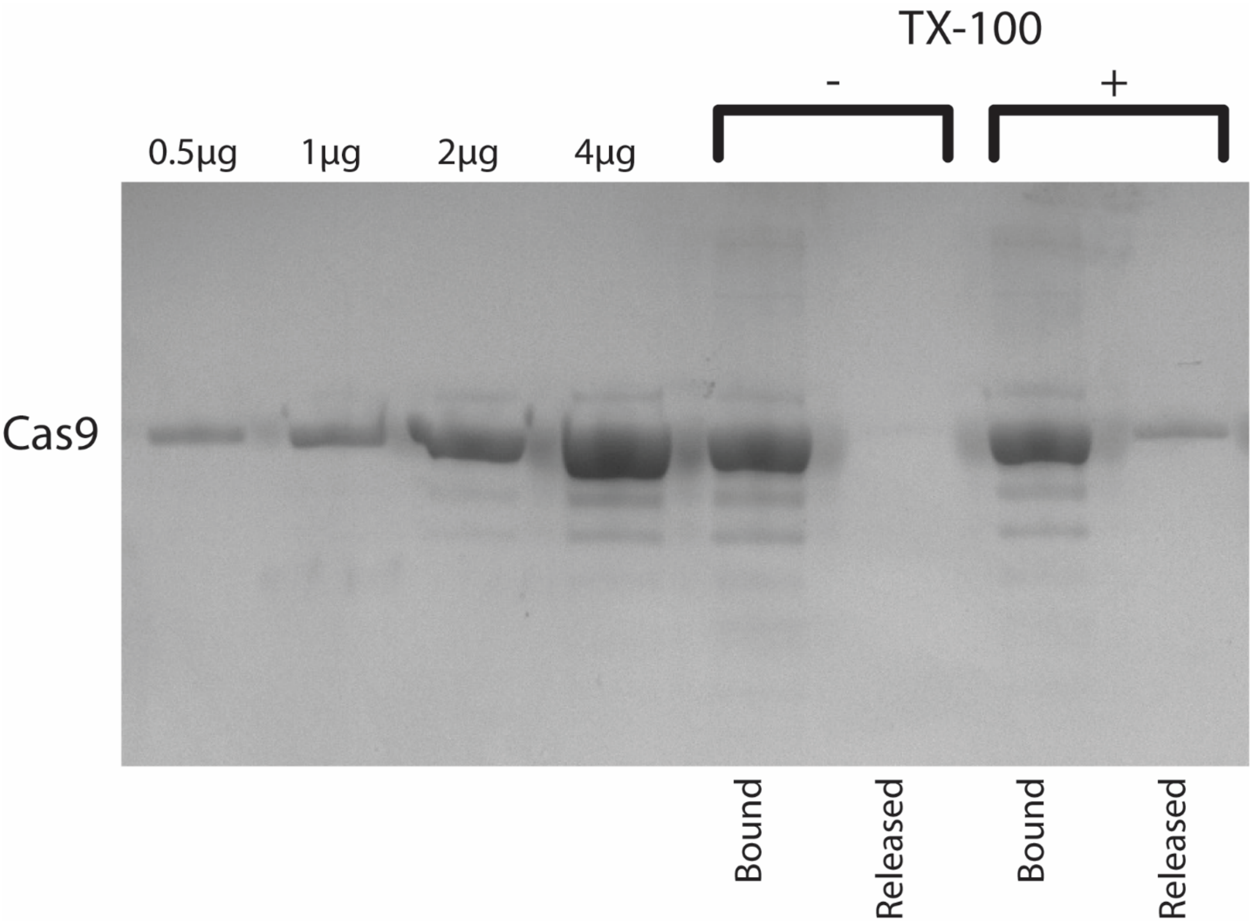

Figure S3. SDS-PAGE electrophoresis visualized by Coomassie stain showing Cas9 present in samples following Triton X-100 treatment of DCD3330 LCMSN.

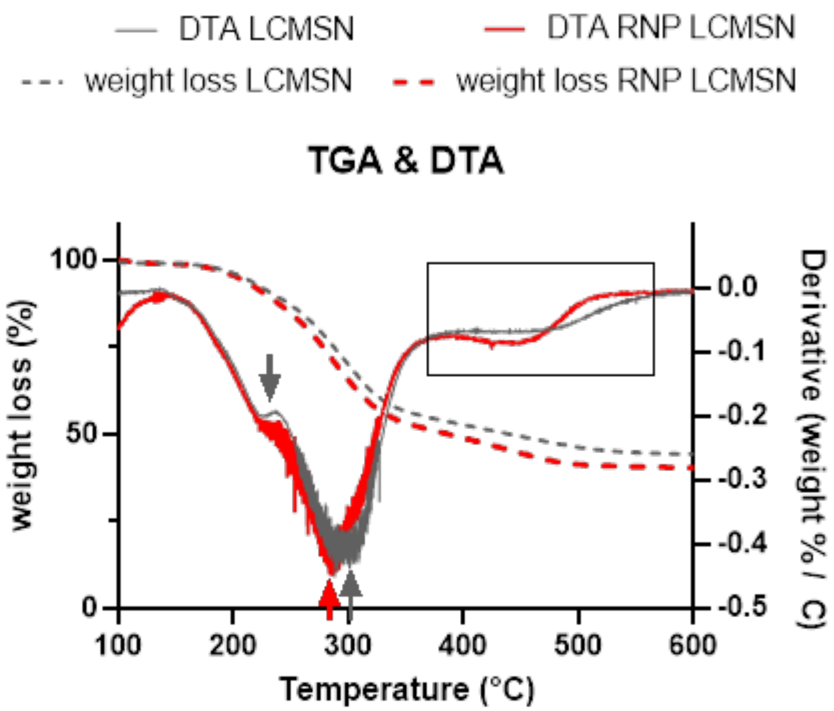

Figure S4: TGA analysis showing the weight loss % profile and its derivative (DTA) in function of temperature for LCMSN and RNP-LCMSN samples. Absolute weight loss is indicated by the dashed lines. RNP degradation can be also detected by DTA curves in 1) the boxed 400-500 C region and 2) the 250-350 C region (red vs grey arrows).

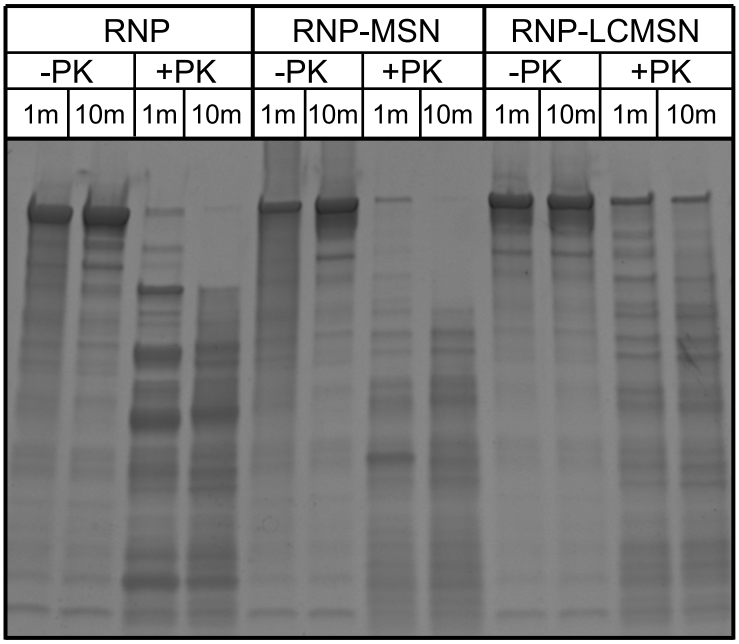

Figure S5. Free RNP, RNP loaded MSN without (MSN-RNP) and with lipid coatings (RNP-LCMSN) were exposed to proteinase K (+PK) for 1 to 10 mins. Cas9 protein was visualized by gel electrophoresis and Coomassie staining.

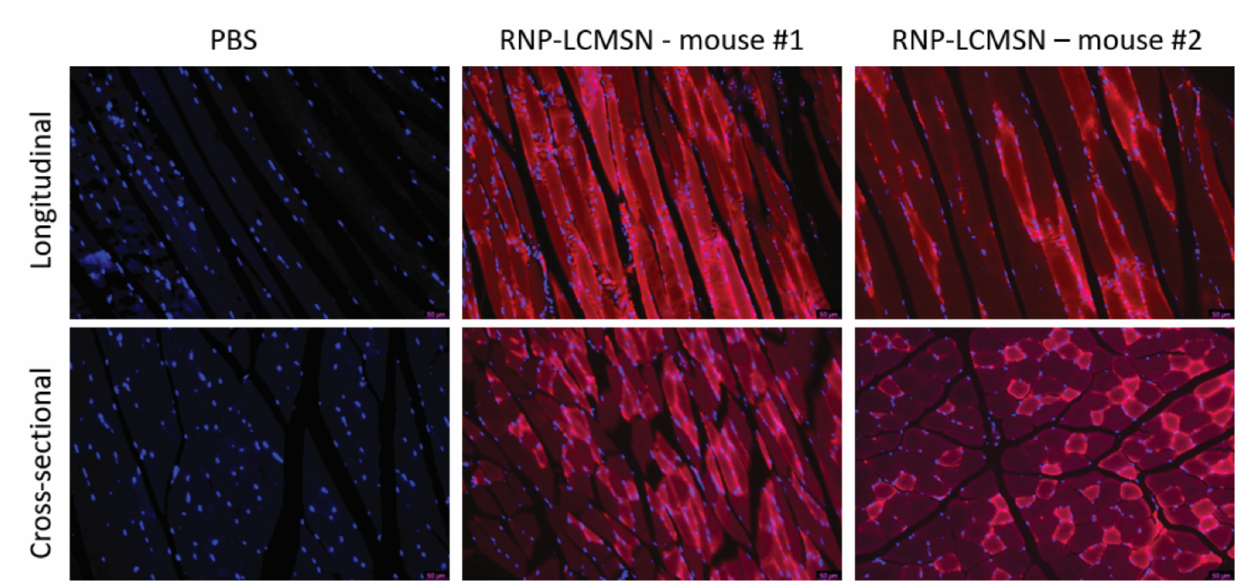

Figure S6. Intramuscular injection into the gastrocnemius muscle of RNP-LCMSNs results in localized delivery and editing in an Ai9 mouse model.

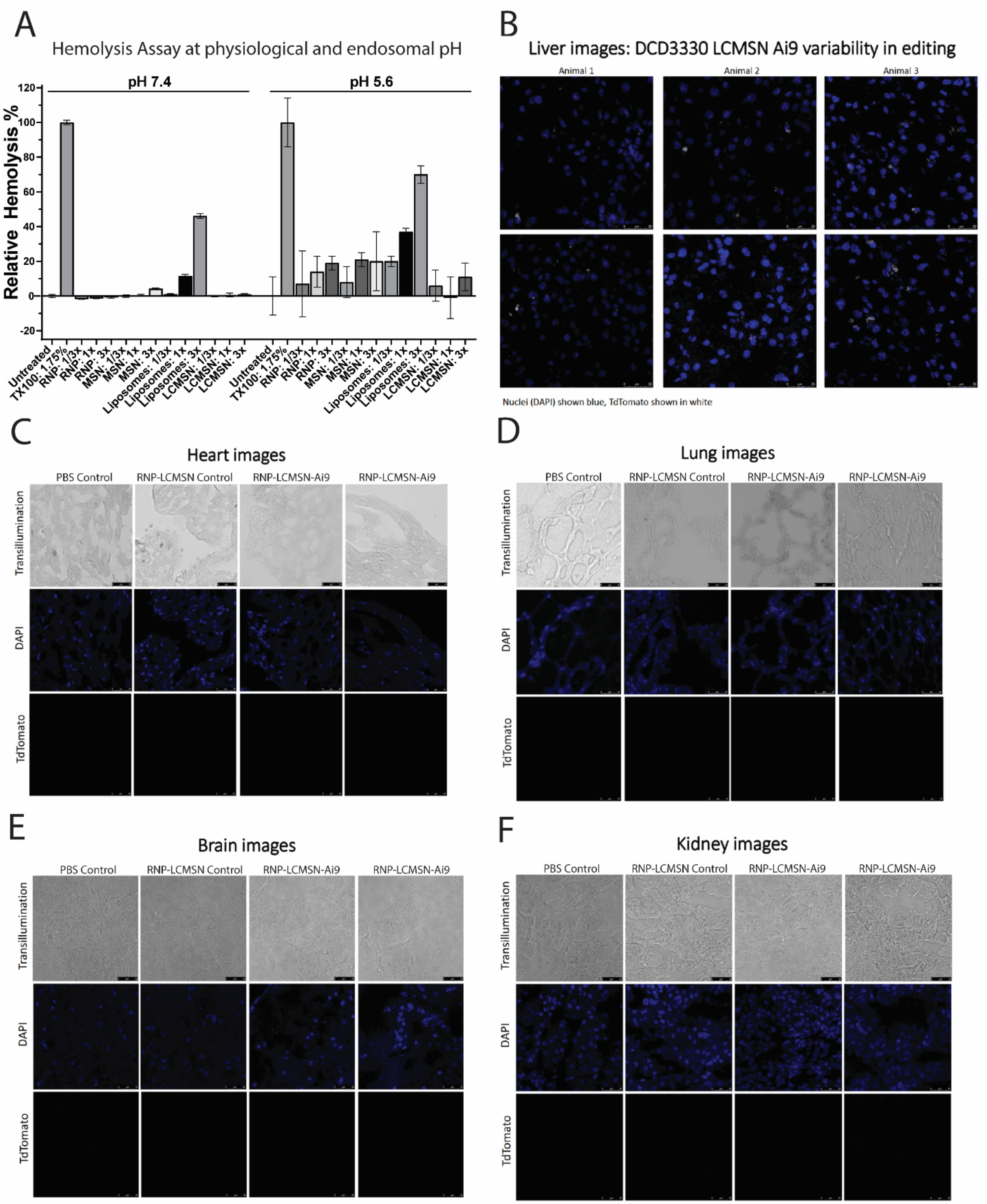

Figure S7. (A) Hemolysis assay performed at physiological pH (pH 7.4) and endosomal pH (pH 5.6). (B-F) Ai9-tdTomato reporter mice (n=3) treated with 1mg of RNP-LCMSN targeting either

the Ai9 stop cassette (RNP-LCMSN-Ai9) or a control sequence (RNP-LCMSN-control) via tail-vein injection. Representative images from all DCD3330 LCMSN Ai9-target treated animals are provided for liver and representative images for each group are provided for all other tissues.

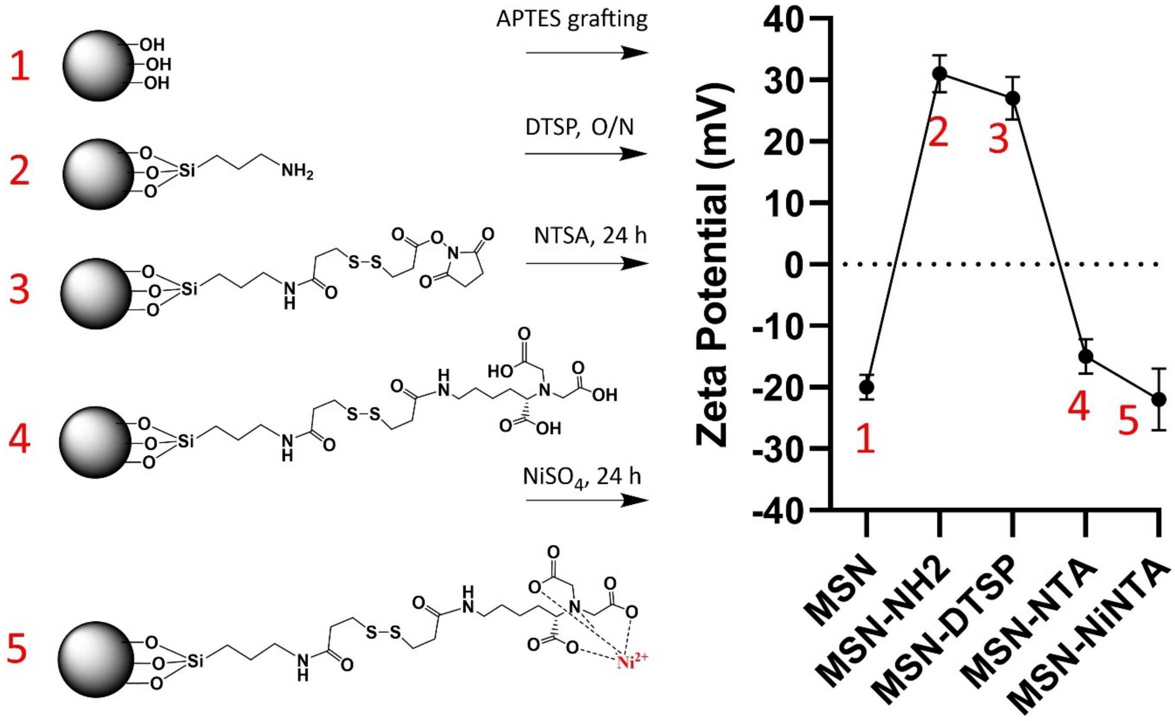

Figure S8. Stellate NiNTA synthesis. Stellate NiNTA particles were synthesized as shown and functionalization was monitored via zeta potential at each step.
